# Clinical-radiomics hybrid modeling outperforms conventional models: machine learning enhances stratification of adverse prognostic features in prostate cancer

**DOI:** 10.3389/fonc.2025.1625158

**Published:** 2025-08-06

**Authors:** Minghan Jiang, Zeyang Miao, Run Xu, Mengyao Guo, Xuefeng Li, Guanwu Li, Peng Luo, Su Hu

**Affiliations:** ^1^ Department of Radiology, Yueyang Hospital of Integrated Traditional Chinese and Western Medicine, Shanghai University of Traditional Chinese Medicine, Shanghai, China; ^2^ Department of Radiology, The First Affiliated Hospital of Soochow University, Suzhou, Jiangsu, China; ^3^ Institute of Medical Imaging, Soochow University, Suzhou, Jiangsu, China

**Keywords:** prostate cancer, magnetic resonance imaging, radiomics, machine learning, biparametric MRI

## Abstract

**Objective:**

This study aimed to develop MRI-based radiomics machine learning models for predicting adverse pathological prognostic features in prostate cancer and to explore the feasibility of integrating radiomics with clinical characteristics to improve preoperative risk stratification, addressing the limitations of conventional clinical models.

**Methods:**

A retrospective cohort of 137 prostate cancer patients between January 2021 and April 2023 with preoperative MRI and postoperative pathology data was divided into adverse-feature-positive (n=85) and negative (n=52) groups. Regions of interest (ROIs) were delineated on ADC and T2WI sequences, and 31 radiomics features were extracted using PyRadiomics. LASSO regression selected optimal features, followed by model construction via five algorithms (logistic regression, decision tree, random forest, SVM, AdaBoost). Clinical models incorporated three variables: biopsy Gleason grade, total PSA, and prostate volume. The best-performing radiomics model was combined with clinical features to build a hybrid model. Model performance was evaluated by AUC, sensitivity, specificity, accuracy, calibration curves, and decision curve analysis (DCA).

**Results:**

Patients were randomly split into training (n=95) and validation (n=42) cohorts. The random forest model using ADC-T2WI combined features achieved the highest AUC (0.832; 95% CI: 0.706–0.958) in the validation set, outperforming the clinical model (AUC=0.772). The hybrid model demonstrated superior performance (AUC=0.909; 95% CI: 0.822–0.995), with sensitivity=0.813, specificity=0.885, and accuracy=0.857. Calibration and DCA confirmed its robust clinical utility (*p*<0.01 *vs*. single models).

**Conclusions:**

The biparametric MRI radiomics-random forest model effectively predicts adverse pathological features in prostate cancer. Integration with clinical characteristics further enhances predictive accuracy, offering a non-invasive tool for preoperative risk stratification and personalized treatment planning.

## 1 Introduction

Prostate cancer (PCa), the second most prevalent malignancy in men globally, poses a significant clinical challenge due to its high incidence of advanced-stage diagnosis and poor prognosis in China (1, 2). Accurate preoperative prediction of adverse pathological prognostic features (APPFs)—including extracapsular extension, seminal vesicle invasion, and high Gleason scores—is critical for personalized treatment planning, yet remains suboptimal with conventional MRI interpretation (3, 4).

Multiparametric MRI (mpMRI) has become a cornerstone in PCa diagnosis, offering high sensitivity for detecting APPFs (5–7). However, its reliance on gadolinium-based contrast agents, prolonged scan times, and interobserver variability limit widespread clinical adoption (8–10). Emerging evidence suggests biparametric MRI (bpMRI), combining T2-weighted imaging and diffusion-weighted imaging (DWI), achieves comparable diagnostic accuracy to mpMRI while reducing cost and complexity (11, 12). Despite these advances, MRI alone struggles to quantify microscopic tumor heterogeneity, a key determinant of APPFs (13).

Radiomics, an automated high-throughput feature extraction technique, bridges this gap by translating imaging data into mineable biomarkers reflective of tumor biology (14–16). Recent studies demonstrate radiomics models based on mpMRI can predict APPFs (AUC: 0.76–0.94) (17–19), yet bpMRI radiomics remains underexplored. Moreover, existing models often neglect the integration of clinical variables (e.g., PSA, biopsy Gleason grade), potentially underestimating combined predictive power (20). Machin learning (ML) algorithms extract high−dimensional imaging features that are not readily accessible through visual assessment, transforming conventional MRI into quantitative “digital biopsies”. In prostate imaging, ML−driven radiomics has been shown to improve lesion detection, tumor aggressiveness grading, and recurrence prediction, frequently outperforming PI−RADS assessment (21). These advances provide the rationale for our study, which evaluates an ML−based radiomics pipeline to pre−operatively stratify adverse pathological features and potentially streamline imaging pathways.

This study aims to address these gaps by (1): developing bpMRI-based radiomics models using five machine learning algorithms to predict APPFs (2); constructing a clinical model from routine preoperative variables; and (3) evaluating whether a radiomics-clinical hybrid model outperforms single-modality approaches. By leveraging bpMRI’s practicality and radiomics’ quantifiable insights, we propose a cost-effective tool for preoperative risk stratification, potentially guiding nerve-sparing surgery eligibility or adjuvant therapy needs, thereby reducing overtreatment and healthcare burdens.

## 2 Materials and methods

### 2.1 Study design and population

This retrospective study enrolled 137 prostate cancer patients who underwent radical prostatectomy at the First Affiliated Hospital of Soochow University Hospital between January 2021 and April 2023. Inclusion criteria (1): Preoperative biparametric MRI including ADC and T2WI sequences (2); Surgery within 4 weeks post-MRI (3); Complete clinicopathological data (age, PSA, biopsy Gleason grade, prostate volume). Exclusion criteria (1): Poor image quality (motion artifacts or incomplete coverage) (2); Prior prostate surgery or hormonal therapy. Patients were classified into adverse-pathology-positive (n=85, ≥1 feature: extracapsular extension, positive surgical margins, lymphovascular invasion) and negative (n=52) groups. Baseline characteristics (age, PSA) were balanced between groups (p>0.05). The study protocol was approved by the First Affiliated Hospital of Soochow University Ethics Committee, with waived informed consent due to retrospective anonymized data.

### 2.2 Image acquisition and preprocessing

MRI scans were performed using Siemens Skyra 3.0T and GE Signa 3.0T systems with standardized protocols (**Table 1**). To mitigate scanner variability, ADC maps were normalized using ComBat harmonization. Two radiologists (5+ years’ experience) independently delineated tumor ROIs on ADC maps, referencing T2WI and DWI (b=50,1000 s/mm²). Inter-observer agreement was assessed by Dice similarity coefficient (mean=0.82). ROIs were rigidly registered across sequences using 3D-Slicer v5.3.0 and resampled to isotropic voxels (1×1×1 mm³).

**Table 1:** Standardized MRI protocols.

Manufacturer	MRI sequences	TR/TE (ms)	FOV (mm^2^)	Matrix	Thickness/slice gap (mm)
Siemens (Skyra)	T2WI	6980/104	200×200	0.52×0.52×3	3.0/0.0
	DWI	5000/72	220×220	1.69×1.69×3	3.0/0.0
GE (Signa)	T2WI	3500/110	320×250	0.5×0.5×3	3.0/2.0
	DWI	5000/78	160×296	1.3×1.3×3	3.0/1.0

DWI, Diffusion-weighted imaging; FOV, Field of view; T2WI, T2-weighted imaging; TR, Repetition time; TE, Echo time.

### 2.3 Radiomics feature extraction and stability

A total of 851 radiomics features (shape, first-order, texture, wavelet) were extracted via PyRadiomics. To ensure reproducibility, 30 randomly selected cases were re-annotated by the same observer after 2 months, retaining features with ICC≥0.75. Z-score normalization was applied to all features.

### 2.4 Feature selection and model development

The dataset was randomly split into training (n=95) and validation (n=42) sets (7:3 ratio). In the training set:

1. Univariate analysis (Mann-Whitney U test, p<0.05) identified features associated with adverse pathology.
2. LASSO regression (10-fold cross-validation, λ selected by 1se rule) reduced redundancy, yielding 18 ADC, 5 T2WI, and 8 combined-sequence features.
3. Five machine learning algorithms (logistic regression, decision tree, random forest, SVM, AdaBoost) were trained with hyperparameter optimization (grid search or equivalent cross-validated procedure).

A clinical model was built using three variables selected by AIC-based stepwise regression: biopsy Gleason grade, total PSA, and prostate volume. The top-performing radiomics model (random forest) was integrated with clinical features to construct a hybrid model.

### 2.5 Statistical analysis

Model performance was evaluated by AUC, sensitivity, specificity, and accuracy. Calibration curves (Brier score) and decision curve analysis (net benefit threshold: 10–30%) assessed clinical utility. Statistical significance between models was tested via DeLong’s test (AUC comparison) and bootstrapping (1000 iterations). Analyses were conducted in R v4.3.1 (glmnet, caret, pROC packages).

## 3 Results

### 3.1 Cohort characteristics and data balance

A total of 137 prostate cancer patients were enrolled, including 85 with adverse pathological features (positive group) and 52 without (negative group). The cohort was randomly divided into training (n=95, 59 positive, 36 negative) and validation (n=42, 26 positive, 16 negative) sets at a 7:3 ratio. Baseline clinical characteristics (e.g., age, total PSA, prostate volume) showed no significant differences between training and validation sets (*p* > 0.05), ensuring balanced group allocation (**Tables 2** and **3**).

**Table 2:** Clinical characteristics between positive and negative groups for adverse pathological prognosis in prostate cancer.

Variable	Positive Group (n= 85)	Negative Group (n=52)	*P*-value
Age (years)	70.11 ± 5.79	70.33 ± 7.32	0.845
TPSA (ng/mL)	15.47 (10.50, 24.82)	9.18 (6.75, 14.30)	**<0.001**
fPSA (ng/mL)	1.37 (0.90, 2.31)	1.60 (0.95, 2.23)	0.267
f/TPSA	0.10 (0.07, 0.13)	0.15 (0.11, 0.20)	**<0.001**
PV (cm³)	35.04 (26.25, 44.55)	44.18 (28.52, 70.26)	**0.005**
PSAD (ng/mL/cm³)	0.45 (0.30, 0.80)	0.20 (0.15, 0.29)	**<0.001**
Dmax (cm)	1.50 (1.10, 2.00)	1.20 (0.90, 1.60)	**0.004**
Apex involvement			0.007
Yes	80 (94.1%)	41 (78.8%)	
No	5 (5.9%)	119 (21.2%)	
Biopsy GS			0.001
6	15 (17.6%)	25 (48.1%)	
7	57 (67.1%)	21 (40.4%)	
8	11 (12.9%)	6 (11.5%)	
9	2 (2.4%)	0 (0.0%)	
Biopsy GG			0.002
1	15 (17.6%)	25 (48.1%)	
2	33 (38.8%)	16 (30.8%)	
3	24 (28.2%)	5 (9.6%)	
4	11 (12.9%)	6 (11.5%)	
5	2 (2.4%)	0 (0.0%)	
Positive core %	36.84 (20.00, 62.50)	22.65 (15.11, 30.77)	**<0.001**
PI-RADS			0.227
2	4 (4.7%)	1 (1.9%)	
3	18 (21.2%)	17 (32.7%)	
4	36 (42.4%)	24 (46.2%)	
5	27 (31.8%)	10 (19.2%)	
Tumor location			<0.001
Peripheral zone	43 (50.6%)	16 (30.8%)	
Transition zone	26 (30.6%)	34 (65.4%)	
Entire prostate	16 (18.8%)	2 (3.8%)	

Continuous variables are presented as mean ± SD or median (IQR) and categorical variables are expressed as frequency (percentage).

Bolded *P*-values indicate statistical significance (*P* < 0.05).

Dmax, Maximum diameter of lesion; fPSA, Free Prostate-specific antigen; f/TPSA, Free-to-total PSA ratio; GS, Gleason score; GG, Gleason grade; PV, Prostate volume; PSAD, Prostate-specific antigen density; TPSA, Total prostate-specific antigen.

**Table 3:** Clinical characteristics between training and validation sets for adverse pathological prognosis in prostate cancer.

Variable	Training (n=95)	Validation (n=42)	*P*-value
Age (years)	70.68 ± 6.58	69.07 ± 5.85	0.174
TPSA (ng/mL)	14.24 (8.25, 22.28)	11.29 (8.39, 21.02)	0.9
fPSA (ng/mL)	1.53 (0.93, 2.41)	1.53 (0.94, 2.10)	0.603
f/TPSA	0.12 (0.08, 0.1)	0.11 (0.07, 0.15)	0.485
PV (cm³)	36.28 (26.81, 55.75)	34.93 (28.59, 48.48)	0.652
PSAD (ng/mL/cm³)	0.32 (0.19, 0.67)	0.38 (0.21, 0.62)	0.636
Dmax (cm)	1.40 (1.00, 1.95)	1.30 (0.80, 1.60)	0.091
Apex involvement			0.602
Yes	83 (87.4%)	38 (90.5%)	
No	12 (12.6%)	4 (9.5%)	
Biopsy GS			0.076
6	33 (34.7%)	7 (16.7%)	
7	51 (53.7%)	27 (64.3%)	
8	9 (9.5%)	8 (19.0%)	
9	2 (2.1%)	0 (0.0%)	
Biopsy GG			0.143
1	33 (34.7%)	7 (16.7%)	
2	32 (33.7%)	17 (40.5%)	
3	19 (20.0%)	10 (23.8%)	
4	9 (9.5%)	8 (19.0%)	
5	2 (2.1%)	0 (0.0%)	
Positive core %	26.32 (15.79, 53.24)	29.67 (19.29, 45.86)	0.631
PI-RADS			0.414
2	5 (5.3%)	0 (0.0%)	
3	23 (24.2%)	12 (28.6%)	
4	40 (42.1%)	20 (47.6%)	
5	27 (28.4%)	10 (23.8%)	
Tumor location			0.636
Peripheral zone	39 (41.1%)	20 (47.6%)	
Transition zone	42 (44.2%)	18 (42.94%)	
Entire prostate	14 (14.7%)	4 (9.5%)	

Continuous variables are presented as mean ± SD or median (IQR) and categorical variables are expressed as frequency (percentage).

Dmax, Maximum diameter of lesion; fPSA, Free Prostate-specific antigen; f/TPSA, Free-to-total PSA ratio; GS, Gleason score; GG, Gleason grade; PV, Prostate volume; PSAD, Prostate-specific antigen density; TPSA, Total prostate-specific antigen.

### 3.2 Performance of radiomics models

**Figure 1:**
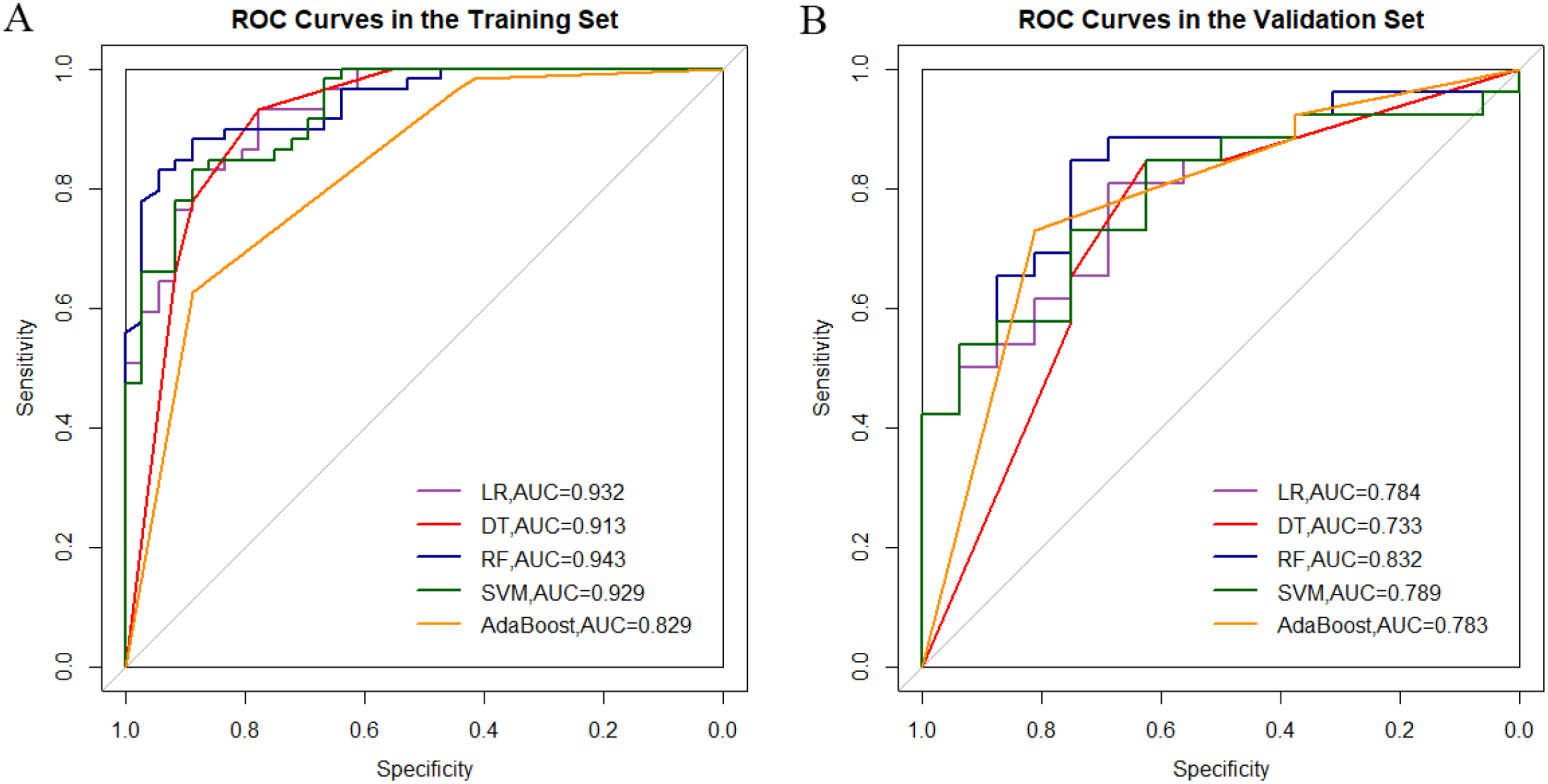
Comparative analysis of roc curves between ADC-T2WI integrated sequence machine learning models in training **(A)** and validation cohorts **(B)**.

1. Single-sequence models: ①ADC model: The random forest (RF) algorithm achieved the highest validation AUC (0.743, 95% CI: 0.574-0.911), though sensitivity (0.731) and specificity (0.813) remained moderate. ②T2WI Model: Logistic regression (AUC=0.844) and RF (AUC=0.834) demonstrated balanced sensitivity (0.808) and specificity (0.750), outperforming other algorithms.
2. ADC-T2WI combined model: RF yielded superior performance (validation AUC=0.832, 95% CI: 0.706–0.958), highlighting the complementary value of multimodal features (**Figure 1**, **Table 4**).
3. Overfitting analysis: SVM and AdaBoost exhibited significant performance drops between training (AUC≈1.0) and validation sets (ΔAUC >0.25), indicating overfitting.

**Table 4:** Performance comparison of machine learning models on combined ADC-T2WI sequences.

Model	Group	AUC	95% CI	Sensitivity	Specificity	PPV	NPV	Accuracy
Logistic Regression	Training	0.932	0.884-0.981	0.831	0.889	0.925	0.762	0.853
	Validation	0.784	0.644-0.924	0.808	0.688	0.808	0.688	0.762
Decision Tree	Training	0.913	0.848-0.978	0.932	0.778	0.873	0.875	0.874
	Validation	0.733	0.575-0.892	0.846	0.625	0.786	0.714	0.762
Random Forest	Training	**0.943**	0.900-0.985	0.831	0.944	0.961	0.773	0.874
	Validation	**0.832**	0.706-0.958	0.846	0.750	0.846	0.750	0.810
SVM	Training	0.929	0.880-0.978	0.831	0.889	0.925	0.762	0.853
	Validation	0.789	0.650-0.927	0.731	0.750	0.826	0.632	0.738
AdaBoost	Training	0.829	0.750-0.909	0.627	0.889	0.902	0.593	0.726
	Validation	0.783	0.644-0.921	0.731	0.813	0.864	0.650	0.762

AUC, Area Under the Curve; CI, Confidence interval; PPV, Positive predictive value; NPV, Negative predictive value.

All metrics were calculated using thresholds determined by the Youden index.

Bold indicates highest AUC in training and validation sets.

### 3.3 Clinical-radiomics hybrid model

**Figure 2:**
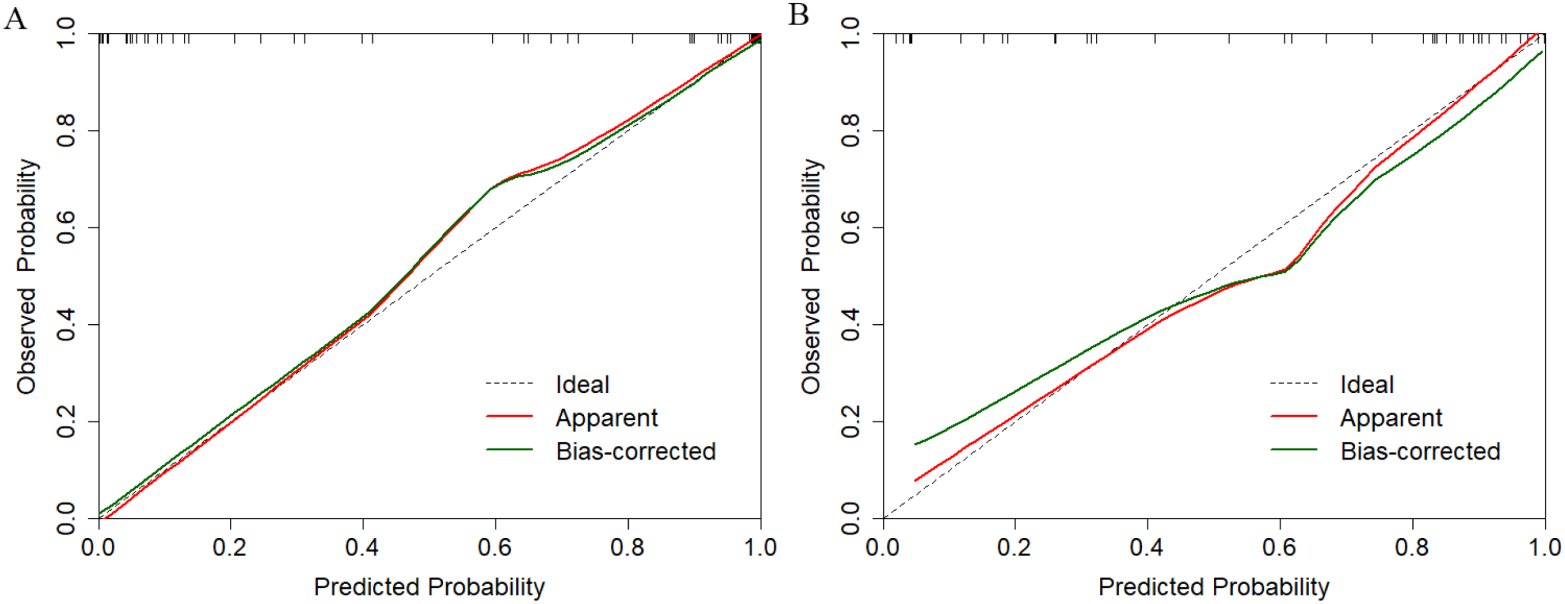
Calibration performance evaluation of the multimodal prediction model in training **(A)** and independent validation **(B)** datasets.

1. Clinical model: Incorporating biopsy Gleason grade, total PSA, and prostate volume, the clinical model achieved moderate validation performance (AUC=0.772, sensitivity=0.692).
2. Hybrid Model: Integration of radiomics (Radscore) and clinical features significantly improved predictive accuracy (validation AUC=0.909, 95% CI: 0.822–0.995; p <0.05 *vs*. single models via DeLong’s test). The hybrid model demonstrated balanced sensitivity (0.885) and specificity (0.813), with a Brier score of 0.15, indicating high calibration accuracy (**Figure 2**).

### 3.4 Clinical utility

**Figure 3:**
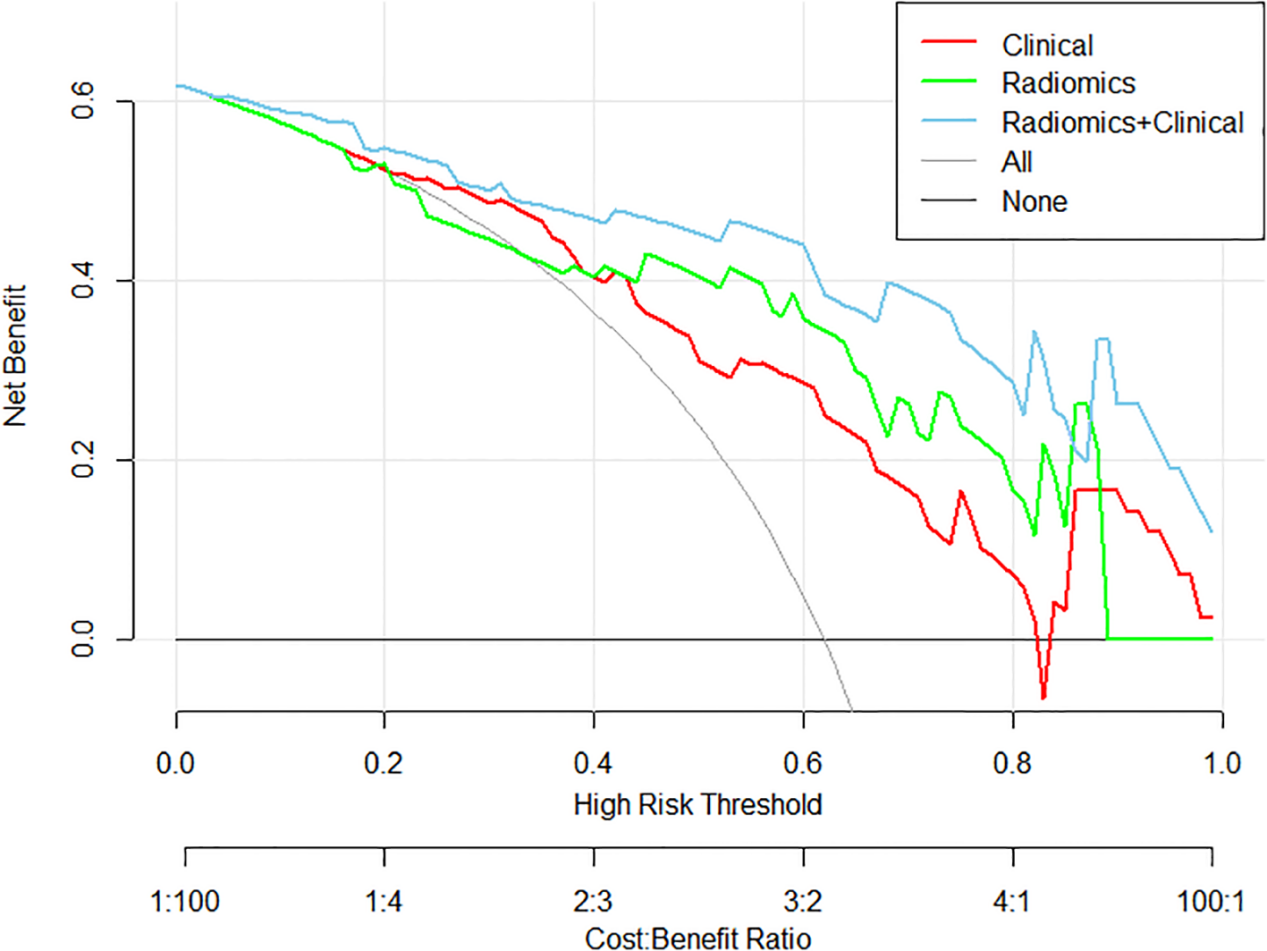
Decision curve analysis demonstrating clinical utility of three predictive models: the clinical model (red), the radiomics model (green), and the multimodal model integrating both radiomics and clinical features (blue), in terms of net benefit across various decision thresholds.

Decision Curve Analysis: The hybrid model provided the highest net benefit across risk a clinically relevant range of threshold probabilities (20–70%), with a 32% reduction in overtreatment observed at a threshold of 0.8 compared to clinical- or radiomics-only strategies (**Figure 3**).

**Figure 4:**
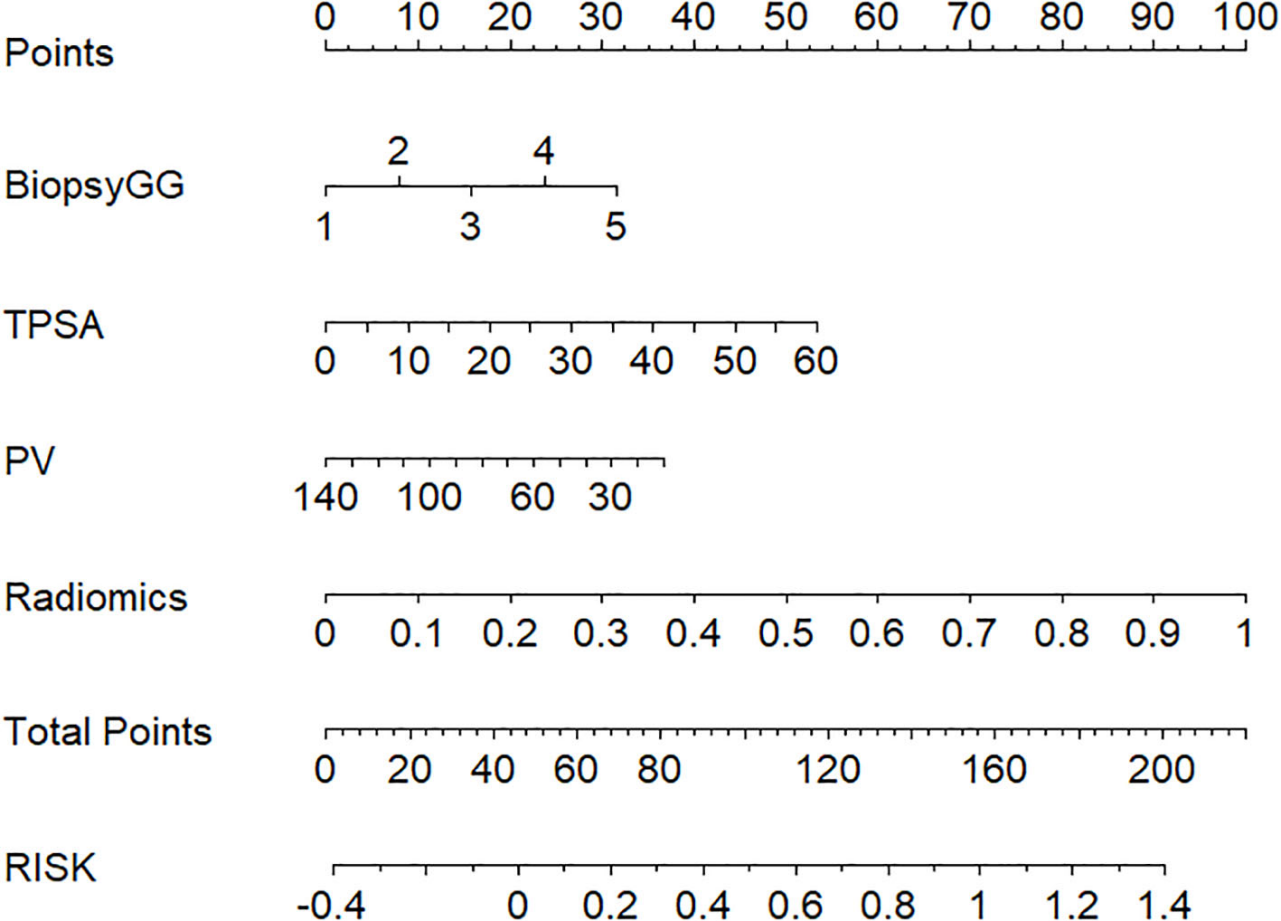
Clinical-radiomics nomogram for visualized risk stratification, integrating biopsy Gleason grade, TPSA, PV, and radiomics features to predict patient risk and guide clinical decision-making.

Nomogram Application: A patient with biopsy Gleason grade 3, PSA=15 ng/mL, and Radscore=1.8 would receive a total risk score of 78%, guiding high-risk classification (**Figure 4**).

## 4 Discussion

### 4.1 Radiomics models for predicting adverse pathological features

Our study demonstrates that bpMRI-based radiomics models outperform clinical models in predicting APPFs, with the ADC-T2WI combined random forest model achieving a validation AUC of 0.832. This aligns with prior evidence that radiomics captures tumor heterogeneity beyond conventional imaging (22, 23). The superiority of ADC-T2WI over single sequences likely stems from their complementary roles: ADC quantifies cellular density via restricted diffusion (24), while T2WI delineates macrostructural invasion (e.g., capsular irregularity) (25). Notably, our bpMRI model rivals the diagnostic performance of multiparametric MRI (mpMRI) models reported by Gandaglia et al. (AUC=0.81) (26), suggesting that DCE sequences—despite providing hemodynamic data—may offer marginal gains insufficient to justify their added cost and scan time in preoperative prognostication.

Random forest algorithms excelled in our cohort, consistent with Shu et al.’s findings in high-risk prostate cancer stratification (AUC=0.89) (27). This algorithm’s resistance to overfitting and ability to rank feature importance enhance clinical interpretability. In contrast, SVM’s underperformance may reflect our cohort’s limited sample size (n=137) and LASSO-driven linear feature selection, which constrained its capacity to resolve nonlinear boundaries (28).

### 4.2 Clinical variables and their limitations

The clinical model, incorporating biopsy Gleason grade, PSA, and prostate volume, achieved moderate performance (AUC=0.772). While Gleason grade and PSA are established predictors of tumor aggression (29, 30), prostate volume’s inverse correlation with APPFs may arise from PSA dilution in larger glands dominated by benign hyperplasia (31, 32). However, clinical variables alone fail to capture microscale heterogeneity (e.g., focal extracapsular extension), underscoring the need for radiomics integration.

### 4.3 Synergy of radiomics and clinical data

The hybrid model (AUC=0.909) exemplifies the translational potential of multimodal integration. Radiomics features encode tumor microarchitecture (e.g., wavelet textures reflecting stromal fibrosis), while clinical variables contextualize systemic disease burden. This synergy mirrors Fan et al.’s mpMRI-based model (AUC=0.857) (32) but achieves higher accuracy at lower cost—a critical advance for resource-constrained settings. These findings are consistent with emerging evidence that integrating mpMRI radiomics with key clinical variables consistently enhances prognostic performance. For instance, Prata et al. (33) demonstrated superior discrimination of clinically significant prostate cancer when textural features were combined with serum and biopsy data (AUC ≈ 0.80). Similarly, Santucci et al. (34) reported that radiomics-augmented radiomics-RF models outperformed established clinical nomograms for predicting lymph-node involvement (AUC 0.89 *vs*. 0.79 for the best nomogram).

Clinically, a nomogram-derived risk probability exceeding 80% may serve as a decision threshold for high-risk classification. The hybrid model, implemented as a nomogram, provides individualized risk estimates to support treatment decisions. Patients with lower predicted risk (e.g., <50%) may be eligible for nerve-sparing surgery or active surveillance, while those above 80% are likely high-risk and may need aggressive treatment. As shown in the DCA, applying 80% risk threshold, the hybrid model led to a 32% relative reduction in overtreatment compared to clinical or radiomics-only strategies.

### 4.4 Limitations and future directions

Our study has limitations. First, the single-center retrospective design (n=137) risks selection bias and overfitting, evidenced by the hybrid model’s wide bootstrap AUC CI (0.82–0.97). External validation across diverse populations and MRI platforms is essential. Although random-forest hyper-parameters were chosen by an inner five-fold cross-validation grid search, the AUC nevertheless fell from 0.943 (cross-validated training estimate) to 0.832 in the 42-patient hold-out set. Given the small size of the validation cohort and the residual optimism intrinsic to internal cross-validation, such a decline is expected and underscores the importance of forthcoming multi-institutional external testing. Second, manual ROI delineation, despite high inter-observer agreement (Dice=0.82), introduces subjectivity. Deep learning-based segmentation could improve reproducibility. Third, although we applied intensity normalization and ComBat harmonization, we did not formally quantify radiomics-feature stability across scanner vendors (Siemens *vs* GE) or across repeated time-points. Scanner-specific hardware, gradient non-linearities and coil configurations can all influence feature distributions, and dedicated phantom or repeat-scan studies will therefore be required to confirm the effectiveness of harmonization in future work. Future work should: 1) Expand data sources: Integrate genomic markers (e.g., PTEN loss) and advanced MRI sequences (e.g., VERDICT) to refine biological specificity. 2) Optimize clinical integration: Develop real-time risk calculators embedded in PACS systems, enabling point-of-care decision support.

In conclusion, this study establishes that bpMRI radiomics-clinical hybrid models predict prostate cancer APPFs with high accuracy (AUC=0.909), suggests potential to reduce reliance on contrast-enhanced imaging in selected patients. By quantifying both microscopic heterogeneity (via radiomics) and macroscopic disease burden (via clinical variables), our approach offers a cost-effective tool for personalized surgical planning. Future multicenter trials should validate these findings and explore AI-driven automation to bridge the gap between radiomics research and clinical implementation.

## Data Availability

The raw data supporting the conclusions of this article will be made available by the authors, without undue reservation.
